# Supplementary material for: Association of sleep duration and sleep quality with hypertension in oil workers in Xinjiang
Source: PeerJ. 2021 May 3;9:e11318. doi: 10.7717/peerj.11318 (PMC8101473; doi:10.7717/peerj.11318)
Supplement: Supplemental Information 2 [file peerj-09-11318-s002.docx]

**Data-Introducing：**

Age：1=“≤ 30 years”，2=“（30-45）years”，3=“﹥45 years”

Gender：1=“Male”，2=“Female”

Sleep quality：1=“Good”，2=“Poor”

Sleep duration：1=“7-8h”，2=“﹤7h”，3=“﹥8h”

Ethnicity：1=“Han”，2=“Others”

Education level：1=“Above senior high school”，2=“Below technical school”

Job title：1=“Above the subtropical high”，2=“Intermediate”，3=“Below the primary level”

Marriage：1=“Unmarried”，2=“Married”，3=“Divorced/Widow/Widower”

Length of service：1=“﹤10years”，2=“（10-20）years”，3=“﹥20 years”

BMI：1=“﹤18.5”，2=“18.5-24”，3=“﹥24”

Smoking：1=“No”，2=“Yes”

Drinking alcohol：1=“No”，2=“Yes”

Income：1=“≥5000￥”，2=“﹤5000”

Shift work：1=“No”，2=“Yes”

Hypertension：1=“No”，2=“Yes”
