# Supplementary material for: Association of sleep duration and sleep quality with hypertension in oil workers in Xinjiang
Source: PeerJ. 2021 May 3;9:e11318. doi: 10.7717/peerj.11318 (PMC8101473; doi:10.7717/peerj.11318)
Supplement: Supplemental Information 3 [file peerj-09-11318-s003.pdf]

匹兹堡睡眠质量指数量表

条目	项目	评分			
		0 分	1 分	2 分	3 分
1	近 1 个月，晚上上床睡觉通常在 |_|_| 点钟				
2	近 1 个月，从上床到入睡通常需要 |_|_|_| min	□≤ 15min	□16~30min	□31~60min	□≥ 60min
3	近 1 个月，通常早上 |_|_| 点起床				
4	近 1 个月，每夜通常实际睡眠 |_|_| h（不等于卧床时间）				
5	近 1 个月，因下列情况影响睡眠而烦恼				
	a.入睡困难（ 30min 内不能入睡）	□无	□＜ 1 次/周	□1~2 次/周	□≥ 3 次/周
	b.夜间易醒或早醒	□无	□＜ 1 次/周	□1~2 次/周	□≥ 3 次/周
	c.夜间去厕所	□无	□＜ 1 次/周	□1~2 次/周	□≥ 3 次/周
	d.呼吸不畅	□无	□＜ 1 次/周	□1~2 次/周	□≥ 3 次/周
	e.咳嗽或鼾声高	□无	□＜ 1 次/周	□1~2 次/周	□≥ 3 次/周
	f.感觉冷	□无	□＜ 1 次/周	□1~2 次/周	□≥ 3 次/周
	g.感觉热	□无	□＜ 1 次/周	□1~2 次/周	□≥ 3 次/周
	h.做噩梦	□无	□＜ 1 次/周	□1~2 次/周	□≥ 3 次/周
	i.疼痛不适	□无	□＜ 1 次/周	□1~2 次/周	□≥ 3 次/周
	j.其它影响睡眠的事情<br>如有，请说明：	□无	□＜ 1 次/周	□1~2 次/周	□≥ 3 次/周
6	近 1 个月，总的来说，您认为您的睡眠质量：	□很好	□较好	□较差	□很差
7	近 1 个月，您用药物催眠的情况：	□无	□＜ 1 次/周	□1~2 次/周	□≥ 3 次/周
8	近 1 个月，您常感到困倦吗？	□无	□＜ 1 次/周	□1~2 次/周	□≥ 3 次/周
9	近 1 个月您做事情的精力不足吗？	□没有	□偶尔有	□有时有	□经常有

计分方法：

成分	内容	评分			
		**0** 分	**1** 分	**2** 分	**3** 分
A、睡眠质量	条目 6 计分	□很好	□较好	□较差	□很差
B、入睡时间	条目 2和 5a 计分累计	□0 分	□1~2 分	□3~4 分	□5~6 分
C、睡眠时间	条目 4 计分	□＞ 7h	□6~7h（不含 6h）	□5~6h（含 6h）	□＜ 5h
D、睡眠效率	以条目 1、3、4 的应答计算睡眠效率 *	□＞ 85%	□75~85%（不含 75%）	□65~75%（含 75%）	□＜ 65%
E、睡眠障碍	条目 5b~5j 计分累计	□0 分	□1~9 分	□10~18 分	□19~27 分
F、催眠药物	条目 7 计分	□无	□＜ 1 次/周	□1~2 次/周	□≥ 3 次/周
G、日间功能障碍	条目 8和 9 的计分累计	□0 分	□1~2 分	□3~4 分	□5~6 分

*睡眠效率计算方法：

$$\text{睡眠效率} = \frac{\text{条目 4(睡眠时间)}}{\text{条目 3(起床时间)} - \text{条目 1(上床时间)}} \times 100\%$$
