# Supplementary material for: Association of sleep duration and sleep quality with hypertension in oil workers in Xinjiang
Source: PeerJ. 2021 May 3;9:e11318. doi: 10.7717/peerj.11318 (PMC8101473; doi:10.7717/peerj.11318)
Supplement: Supplemental Information 4 [file peerj-09-11318-s004.pdf]

Pittsburgh Sleep Quality Index

Entry	Item		Scoring			
			0	1	2	3
1	During the past month, when have you usually gone to bed at night? ____________					
2	During the past month, how long has it usually takes you to fall asleep each night?		☐≤ 15 minutes	☐16~30 minutes	☐ 31~60 minutes	☐ ≥ 60 minutes
3	During the past month, when have you usually gotten up in the morning? ____________					
4	During the past month, how many hours of actual sleep did you get at night? (This may be different than the number of hours you spend in bed.) ___________					
5	During the past month, you have been troubled by the following conditions that affect your sleep:					
	a. Difficulty in falling asleep (Cannot get to sleep within 30 minutes)		☐Not during the past month	☐Less than once a week	☐Once or twice a week	☐Three or more times a week
	b. Wake up in the middle of the night or early morning		☐Not during the past month	☐Less than once a week	☐Once or twice a week	Three or more times a week
	c. Have to get up to use the bathroom		☐Not during the past month	☐Less than once a week	☐Once or twice a week	☐Three or more times a week
	d. Cannot breathe comfortably		☐Not during the past month	☐Less than once a week	☐Once or twice a week	☐Three or more times a week
	e. Cough or snore loudly		☐Not during the past month	☐Less than once a week	☐Once or twice a week	☐Three or more times a week
	f. Feel too cold		☐Not during the past month	☐Less than once a week	☐Once or twice a week	☐Three or more times a week
	g. Feel too hot		☐Not during the past month	☐Less than once a week	☐Once or twice a week	☐Three or more times a week
	h. Had bad dreams		☐Not during the	☐Less than once	☐Once or	☐Three or more

			past month	a week	twice a week	times a week
	i. Have pain		☐Not during the past month	☐Less than once a week	☐Once or twice a week	☐Three or more times a week
	j. Other reasons, please describe: How often during the past month have you had trouble sleeping because of this?		☐Not during the past month	☐Less than once a week	☐Once or twice a week	☐Three or more times a week
6	During the past month, how would you rate your sleep quality overall?		☐Very good	☐Fairly good	☐Fairly bad	☐Very bad
7	During the past month, how often have you taken medicine to help you sleep?		☐Not during the past month	☐Less than once a week	☐Once or twice a week	☐Three or more times a week
8	During the past month, how often have you had trouble staying awake while driving, eating meals, or engaging in social activity?		☐Not during the past month	☐Less than once a week	☐Once or twice a week	☐Three or more times a week
9	During the past month, how much of a problem has it been for you to keep up enough enthusiasm to get things done?		☐No problem at all	☐Only a very slight problem	☐Somewhat of a problem	☐A very big problem

Scoring method:

Entry	Content		Scoring			
			0	1	2	3
Sleep quality	6		□Very good	□Fairly good	□Fairly bad	□Very bad
Sleep latency	2+5a		□0	□1~2	□3~4	□5~6
Sleep duration	4		□>7h	□6~7h (excluding 6h)	□5~6 (including 6h)	□<5h
Sleep efficiency	1, 3, 4		□>85%	□75~85% (excluding 75%)	□65~75% (including 75%)	□<65%
Sleep disturbance	5b+5c+5d+5e+5f+5g+5h+5i+5j		□0	□1~9	□10~18	□19~27
Sleeping medication	7		□Not during the past month	□Less than once a week	□Once or twice a week	□Three or more times a week
Daytime dysfunction	8+9		□0	□1~2	□3~4	□5~6

* Sleep efficiency is calculated by

$$\text{Sleep efficiency} = \frac{4(\text{sleep disturbance})}{3(\text{wake up time}) - 1(\text{bedtime})} \times 100\%$$
