# Supplementary material for: Association of sleep duration and sleep quality with hypertension in oil workers in Xinjiang
Source: PeerJ. 2021 May 3;9:e11318. doi: 10.7717/peerj.11318 (PMC8101473; doi:10.7717/peerj.11318)
Supplement: Supplemental Information 5 [file peerj-09-11318-s005.doc]

**石油工人职业健康状况调查表**

**尊敬的先生（女士）：**

为了能对我市石油野外作业人员的职业紧张、身心健康状况及卫生服务需求有一个全面了解，以便为您提供高质量、高效率的卫生服务，我们向您作此调查！希望您能耐心、如实的回答以下问题，感谢您在百忙之中的参与和配合！

**个人基本信息**

1、您的姓名_________________________________

2、您的年龄__________岁

3、您的性别：①男 ②女

4、您的族别：①汉族 ②维吾尔族 ③哈萨克族 ④回族 ⑤蒙古族 ⑥其他___________

您的文化程度：①初中 ②高中 ③中专 ④大专 ⑤本科 ⑥研究生及以上

6、您的工种：①钻井 ②录井 ③井下作业 ④试油 ⑤测井 ⑥输油

⑦采油 ⑧炼化工种 ⑨其他__________

7、您的工作年限__________年

8、倒班情况：①固定白班 ②两班倒 ③三班两倒 ④四班三倒 ⑤其他____

8、您的职称：①无 ②初级 ③中级 ④副高级及高级

9、您的婚姻状况：①未婚 ②已婚 ③离异 ④丧偶

10、您的月均收入__________元

11、您的身高___________cm 体重___________kg

13、您是否患有高血压？①是 ②否

14、您是否吸烟？①经常吸 ②偶尔吸 ③不吸

15、您是否饮酒？①经常饮 ②偶尔饮 ③不饮
