# Supplementary material for: Association of sleep duration and sleep quality with hypertension in oil workers in Xinjiang
Source: PeerJ. 2021 May 3;9:e11318. doi: 10.7717/peerj.11318 (PMC8101473; doi:10.7717/peerj.11318)
Supplement: Supplemental Information 6 [file peerj-09-11318-s006.doc]

**Oil Workers Occupational Health Survey**

**Dear Mr. (lady):**

In order to be able to have a comprehensive understanding of the occupational stress, physical and mental health and health services of the petroleum field workers in our city, to provide you with high quality, high-efficiency health services, we have to make this survey to you! I hope you can patiently answer the following questions, thank you for your participation and cooperation in your busy schedule!

**Personal information**

1. What is your name _____________________

2. How old are you __________

3. What is your gender： ① Male ② Female

4. What is your ethnicity: ① Han ② Uyghur ③ Kazakh ④ Hui nationality ⑤Mongolian

⑥ Other___________

5. What is your degree of education：① Junior middle school ② High school ③Technical secondary school ④ Junior college ⑤ Undergraduate ⑥ Graduate and above

6. What is your work：① Drilling ② Well log ③ Underground operation ④ Test oil ⑤ Well logging ⑥ Oil transportation ⑦ oil extraction ⑧ Refining and chemical work ⑨ Other___________

7. How many years have you worked__________

8. Shift situation：① Regular day shift ② Two shifts ③Two shifts in three shifts ④ Three shifts in four shifts. ⑤ Other____

9. What's your job title：① No ② Primary ③ Intermediate ④ Deputy senior and senior

10. What is your current marital status：① Unmarried ② Married ③ Divorced ④Bereaved a spouse

11. How much is your monthly income__________

12. How tall are you ___________cm How much do you weigh ___________kg

13. Do you have high blood pressure ① Yes ② No

14. Do you smoke ① Often ② Occasionally ③ No

15. Do you drink alcohol ① Often ② Occasionally ③ No
